# Total intravenous anesthesia decreases hospital stay but not incidence of postoperative pulmonary complications after lung resection surgery: a propensity score matching study

**DOI:** 10.1186/s12871-023-02260-4

**Published:** 2023-10-17

**Authors:** Fu-Kai Hsu, Hung-Wei Cheng, Wei-Nung Teng, Po-Kuei Hsu, Han-Shui Hsu, Wen-Kuei Chang, Chien‑Kun Ting

**Affiliations:** 1 Department of Anesthesiology, Taipei Veterans General Hospital, No. 201, Sec. 2, Shih-pai Rd, 11217 Taipei, Taiwan; 2 School of Medicine, National Yang Ming Chiao Tung University, Taipei, Taiwan; 3 Institute of Biomedical Informatics, National Yang Ming Chiao Tung University, 112 Taipei, Taiwan, ROC; 4 Department of Surgery, Division of Thoracic Surgery, Taipei Veterans General Hospital, Taipei, Taiwan; 5 Institute of Emergency and Critical Care Medicine, National Yang Ming Chiao Tung University, 112304 Taipei, Taiwan

**Keywords:** Anesthesia, Postoperative pulmonary complications, Lung resection surgery, Volatile anesthesia, Total intravenous anesthesia

## Abstract

**Background:**

There is no consensus regarding the superiority of volatile or total intravenous anesthesia (TIVA) in reducing the incidence of postoperative pulmonary complications (PPCs) after lung resection surgery (LRS). Thus, the aim of this study was to investigate the different anesthetic regimens and the incidence of PPCs in patients who underwent LRS. We hypothesized that TIVA is associated with a lower incidence of PPCs than volatile anesthesia.

**Methods:**

This was a retrospective cohort study of patients who underwent LRS at Taipei Veterans General Hospital between January 2016 and December 2020. The patients’ charts were reviewed and data on patient characteristics, perioperative features, and postoperative outcomes were extracted and analyzed. The patients were categorized into TIVA or volatile anesthesia groups and their clinical data were compared. Propensity score matching was performed to reduce potential selection bias. The primary outcome was the incidence of PPCs, whereas the secondary outcomes were the incidences of other postoperative events, such as length of hospital stay (LOS) and postoperative nausea and vomiting (PONV).

**Results:**

A total of 392 patients each were included in the TIVA and volatile anesthesia groups. There was no statistically significant difference in the incidence of PPCs between the volatile anesthesia and TIVA groups. The TIVA group had a shorter LOS (*p* < 0.001) and a lower incidence of PONV than the volatile anesthesia group (4.6% in the TIVA group vs. 8.2% in the volatile anesthesia group; *p* = 0.041). However, there were no significant differences in reintubation, 30-day readmission, and re-operation rates between the two groups.

**Conclusions:**

There was no significant difference between the incidence of PPCs in patients who underwent LRS under TIVA and that in patients who underwent LRS under volatile anesthesia. However, TIVA had shorter LOS and lower incidence of PONV which may be a better choice for maintenance of anesthesia in patients undergoing LRS.

**Supplementary Information:**

The online version contains supplementary material available at 10.1186/s12871-023-02260-4.

## Background

Lung cancer is the most common cause of cancer-related deaths, accounting for 1.8 million deaths each year [1]. In Taiwan, the number of lung cancer surgeries performed in 2020 was three times that recorded in 2010, most likely owing to the increasing use of low-dose computed tomography in lung cancer screening [2]. In addition, the incidence of postoperative pulmonary complications (PPCs) after lung resection surgery (LRS) was from < 1–23% [3]. PPCs are associated with higher mortality rate, longer length of hospital stay (LOS), and increased healthcare costs [3]. Thus, it is important to investigate the possible protective factors against PPCs in patients undergoing LRS to improve their clinical outcomes.

General anesthesia is usually induced before LRS, and the most common anesthesia regimens used include volatile anesthesia, which involves the administration of volatile anesthetics such as sevoflurane or desflurane, and total intravenous anesthesia (TIVA), which involves the administration of intravenous anesthetic agents such as propofol. Previous studies have shown that TIVA has a weaker effect on hypoxic pulmonary vasoconstriction (HPV), which is related to hypoxemia during one-lung ventilation (OLV), than volatile anesthesia [4]. Moreover, TIVA appears to associate with a lower overall mortality rate after cancer surgery than volatile anesthesia [5]. Some studies have revealed that volatile anesthetics diminish both pulmonary and systemic inflammatory responses and reduce the expression of proinflammatory cytokines [6]. Volatile anesthetics can also protect major organs from ischemia/reperfusion tissue damage [7]. Despite these reported findings regarding the effects of TIVA and volatile anesthesia on perioperative outcomes, there is no consensus on the superiority of volatile or total intravenous anesthesia (TIVA) in reducing the incidence of PPCs after LRS. Therefore, the choice of the regimen used for maintenance of anesthesia is usually based on hospital policy or the anesthesiologist’s preference. Thus, the aim of this study was to explore the association between different anesthetic regimens and the incidence of PPCs in patients who underwent LRS. Based on the results of previous studies, we hypothesized that TIVA might be associated with a lower incidence of PPCs in patients who underwent LRS than volatile anesthesia.

## Materials and methods

### Study design and patient selection

This was a retrospective cohort study conducted to find out the association between different anesthetic regimens and the incidence of PPCs in patients who underwent LRS. This study was approved by the Taipei Veterans General Hospital Institutional Review Board (IRB-TPEVGH no.: 2021-03-003CC). The review board waived the need for patient consent. All methods were conducted according to the local guidelines and regulations of Taipei Veterans General Hospital.

We reviewed the electronic medical database of our institution and extracted the data of all patients who underwent video-assisted thoracoscopic surgery (VATS) at our medical center between January 2016 and December 2020. Patients who met the following criteria were excluded from the analysis: [1] missing relevant data, such as demographic information, surgical and anesthetic features, or postoperative outcomes; [2] did not undergo LRS; [3] underwent thoracotomy or intraoperative conversion to open resection; [4] underwent tubeless surgery; [5] underwent delayed extubation; and [6] an American Society of Anesthesiologists (ASA) class four or higher pre-anesthesia health status. The included patients were categorized in two groups: the TIVA group, which comprised patients who received intravenous anesthetics for the maintenance of general anesthesia, and the volatile anesthesia group, which included those who received volatile anesthetics for the maintenance of general anesthesia.

### Anesthesia management

Bispectral index (BIS) (Medtronic, Minneapolis, MN) monitoring and hemodynamic monitoring with electrocardiography, pulse oximetry, and noninvasive and invasive arterial blood pressure measurements were routinely performed for the evaluation of patients in both groups. In the volatile anesthesia group, 1–3 ug of fentanyl per kilogram of body weight and 1-2.5 mg of 1% propofol per kilogram of body weight were administered for induction of general anesthesia. Volatile anesthetics, such as sevoflurane or desflurane, were used for maintenance of anesthesia. In the TIVA group, propofol and remifentanil were continuously infused using a target-controlled infusion system based on the Schnider and Minto models, respectively. The doses of anesthetics were adjusted to maintain the BIS between 40 and 60. The intercostal block with 3–5 mL 0.5% bupivacaine for each level was performed by the surgeon in the end of the surgery. Parecoxib 40 mg was administrated every 12 h from the beginning of the surgery to the 24–48 h after surgery and Ultracet tablets was used for rescue analgesia.

### Data collection

We extracted the following data from patients’ records: age, sex, height, weight, ASA class, preoperative ratio of the forced expiratory volume in the first one second to the forced vital capacity of the lungs (FEV1/FVC) [6], and underlying disease (myocardial infarction, congestive heart failure, peripheral vascular disease, cerebrovascular accident or transient ischemic attack, dementia, chronic obstructive pulmonary disease, connective tissue disease, peptic ulcer disease, liver disease, diabetes mellitus, hemiplegia, chronic kidney disease, solid tumor, lymphoma, leukemia, acquired immune deficiency syndrome) assessed using Charlson comorbidity index (supplementary file 1) [8]. Intraoperative data, such as anesthesia regimens, anesthesia time were recorded. Postoperative events, such as PPCs (respiratory failure, respiratory infection, atelectasis, pneumothorax, bronchospasm, pleural effusion, upper airway obstruction, prolonged air leakage, pulmonary embolism,) [3, 6, 9], subcutaneous emphysema, chylothorax, re-operation, reintubation, LOS, 30-day unplanned readmission, and postoperative nausea and vomiting (PONV), were recorded as well. PONV was defined as any nausea or vomiting occurring during the first 24 to 48 h after the surgery [10]. The primary outcome was the difference in the incidence of PPCs between the TIVA and volatile anesthetic groups. The secondary outcomes were the differences in the incidences of other postoperative events (subcutaneous emphysema, chylothorax, re-operation, reintubation, LOS, 30-day unplanned readmission, and PONV) between the two groups.

### Statistical analysis

Comparisons of the baseline characteristics of the patients in the volatile anesthesia and TIVA groups were performed using the independent t-test or Wilcoxon rank-sum test for continuous variables and the chi-square test for categorical variables, as appropriate. To eliminate imbalances in the collected covariates of two groups, propensity score matching (PSM) was performed with 1:1 nearest neighbor matching method and caliper value of 0.1(supplementary file 2). The covariates included age, height, weight, gender, ASA classification, FEV1/FVC ratio, CCI, anesthesia time and blood loss. Statistical significance was set at P < 0.05 (two-tailed). Based on the previous study by Lee et al. [9], we estimated the minimum requirement of sample size was 264 to achieve a power of 0.8 given a type I error rate of 0.05 [11]. To compare the difference between two groups by the time until discharge, we performed the Cox regression analysis after confirming the proportional hazard assumption. The postoperative deaths were excluded from the analysis. All statistical analyses were performed using Statistical Package for the Social Sciences (SPSS) 28.0 (IBM Corp., Armonk, NY, USA) software.

## Results

### Patient characteristics

**Fig. 1:**
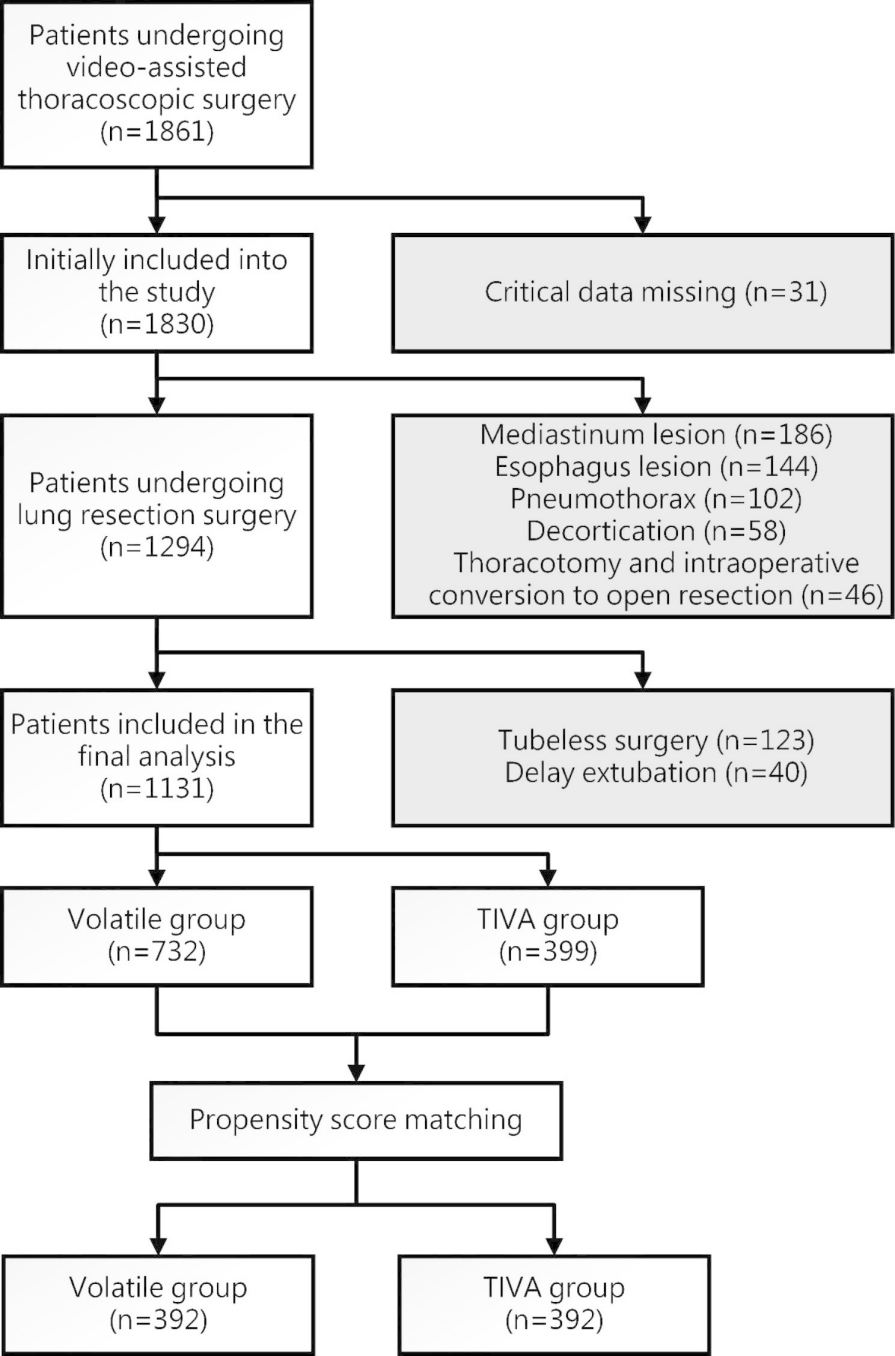
Flow diagram for patient selection

A total of 1861 patients who underwent VATS at our hospital between January 2016 and December 2020 were screened for inclusion into this study. Of these, 730 patients were excluded from the analysis based on the exclusion criteria (Fig. 1). Thus, 1131 patients were included for analysis, with 732 patients in the volatile anesthesia group and 399 patients in the TIVA group. After PSM, 392 patients from each group were included in the matched TIVA and volatile anesthesia groups. The baseline characteristics of the matched groups are shown in Table 1.

**Table 1:** Patient characteristics

	Original data				After PSM		
	TIVA group (n = 399)	Volatile anesthesia group (n = 732)	SMD		TIVA group (n = 392)	Volatile anesthesia group (n = 392)	SMD
Age (year)	57.9 ± 13.3	59.7 ± 13.7	-0.140		58.2 ± 13.1	58.2 ± 14.1	0.004
Height (cm)	162.1 ± 9.9	160.9 ± 8.9	0.117		161.6 ± 8.3	162.1 ± 9.2	-0.048
Weight (kg)	63.2 ± 12.0	62.6 ± 11.8	0.045		63.0 ± 11.9	63.3 ± 12.3	-0.023
BMI (kg/m^2^)	24.0 ± 3.9	24.1 ± 3.6			24.1 ± 3.8	24.0 ± 3.7	
Sex (female)	230(57.6%)	427(58.3)	0.014		229(58.4%)	224(57.1%)	-0.026
ASA classification			-0.027				0.020
I	32(8.0%)	66(9.0%)			32(8.2%)	39(9.9%)	
II	286(71.7%)	503(68.7%)			281(71.7%)	271(69.1%)	
III	81(20.3%)	163(22.3%)			79(20.2%)	82(20.9%)	
FEV1/FVC (%)	80.4 ± 8.1	80.5 ± 8.2	-0.002		80.4 ± 8.0	80.6 ± 7.8	-0.022
CCI	4(3–6)	4(3–6)	-0.108		4(3–6)	4(3–6)	0.033
Anesthesia time(mins)	187.5(150–240)	165(135–240)	-0.215		165.0(135–240)	180.0(135–240)	-0.042
Blood loss (mL)	30.0(30–650)	30.0(10-5450)	-0.763		30.0(30–250)	30.0(10–250)	-0.048

Values are presented as mean ± SD, counts (percent), or median (IQR). Blood loss was presented as median (range)

Abbreviations: ASA, American Society of Anesthesiologists; BMI, body mass index; CCI, Charlson Comorbidity Index; FEV1/FVC, ratio of the forced expiratory volume in the first second to the forced vital capacity; IQR, interquartile range; PSM, propensity score matching; SMD, standardized mean difference; TIVA, total intravenous anesthesia;

### Postoperative outcomes

**Fig. 2:**
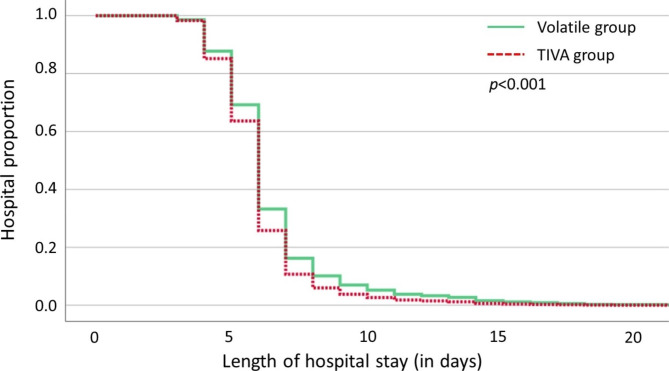
Cox proportional hazards regression model for length of hospital stay in the volatile anesthesia and TIVA groups

After PSM, the incidence of PPCs between the two groups was not statistically significant. In addition, there was no significant difference in 30-day unplanned readmission and re-operation between the two groups. One re-intubation event was happened in the volatile anesthesia group due to respiratory failure caused by aspiration pneumonia. Two patients in the volatile anesthesia group underwent re-operation. One was due to a prolonged air leak; thoracoscopic wedge resection was performed as well and the other was due to torsion of left upper lobe; emergent left upper lobe anterior and lingula segmentectomy was performed. TIVA group had a shorter LOS than the volatile anesthesia group, and the Cox regression model showed a significant difference between the two groups (*p* < 0.001; Fig. 2). The incidence of PONV in TIVA group was lower than that in the volatile anesthesia group (4.6% vs. 8.2%, *p* = 0.041). The postoperative outcomes of the volatile anesthesia and TIVA groups are shown in Table 2.

**Table 2:** Comparison of postoperative pulmonary complications and other outcomes in the volatile anesthesia and TIVA groups after propensity score matching

Variables	TIVA group (n = 392)	Volatile anesthesia group (n = 392)	*p*-value
PPCs	23(5.9%)	26(6.6%)	0.658
Respiratory failure	0	2(0.5%)	0.157
Respiratory infection	1(0.3%)	3(0.8%)	0.316
Atelectasis	3(0.8%)	1(0.3%)	0.316
Pneumothorax	10(2.6%)	5(1.3%)	0.192
Bronchospasm	1(0.3%)	0	0.317
Pleural effusion	2(0.5%)	6(1.5%)	0.155
Upper airway obstruction	0	0	-
Prolonged air leakage	6(1.5%)	9(2.3%)	0.434
Pulmonary embolism	0	0	-
30-day unplanned readmission	4(1.0%)	7(1.8%)	0.362
Reintubation	0	1(0.3%)	0.317
Length of hospital stay	6(3–24)	6(3–32)	< 0.001
PONV	18(4.6%)	32(8.2%)	0.041
Re-operation	0	2(0.5%)	0.157
Subcutaneous emphysema	3(0.8%)	7(1.8%)	0.203
Chylothorax	6(1.5%)	8(2.0%)	0.590

Values are presented as median (range) or counts (percent)

Abbreviations: PONV, postoperative nausea and vomiting; PPCs, postoperative pulmonary complications; TIVA, total intravenous anesthesia

## Discussion

In this study, we investigated the association between different anesthetic regimens and the incidence of PPCs in patients who underwent LRS. We used PSM to reduce imbalances between the TIVA and volatile anesthesia groups and to obtain a more reliable estimate of the relationship between anesthesia regimens and the incidence of PPCs [12]. In addition, the Charlson comorbidity index was considered in this study because the presence of a comorbidity is an important predictor of PPCs [3, 13]. However, the results of this study did not support our hypothesis that TIVA is associated with a lower incidence of PPCs than volatile anesthesia.

The pathophysiology of PPCs is complex. Changes in the respiratory system after anesthesia, residual atelectasis, abnormal respiratory control, and ineffective coughing all contribute to the occurrence of PPCs [3]. HPV is important for maintaining oxygenation after induction of anesthesia. TIVA has a weaker effect on HPV than volatile anesthesia, which is an advantage during OLV [4]. Moreover, patients who underwent surgery under TIVA show a lower incidence of postoperative cognitive dysfunction, which is a non-modifiable patient factor related to PPCs, than those who received volatile anesthetics [3, 14]. In contrast, volatile anesthetics have anti-inflammatory effects, and patients undergoing LRS under volatile anesthesia show lower levels of pro-inflammatory cytokines [6]. Inflammatory response to surgery may impair the activities of the respiratory muscle group, which then leads to the occurrence of PPCs [3]. In a previous retrospective study, no statistically significant difference in the incidence of PPCs between the volatile anesthesia and TIVA groups was observed; however, prolonged air leak occurred more frequently in the volatile anesthesia group. This difference between the two groups may have been caused by a change in postoperative care strategy rather than the anesthesia regimens used [9]. In a randomized controlled trial of patients who underwent LRS with prolonged OLV, the TIVA group showed a higher incidence of PPCs and higher 1-year mortality than the volatile anesthesia group [6]. However, it should be noted that the duration of OLV is a risk factor for PPCs. In a meta-analysis, patients who underwent cardiac surgery under TIVA showed a higher incidence of PPCs than those who underwent the surgery under volatile anesthesia; however, there was no significant difference between the patients who underwent non-cardiac surgery under volatile anesthesia and those who underwent the surgery under TIVA. The difference between cardiac and non-cardiac surgeries may contribute to the cardioprotective effects of volatile anesthetics, and the beneficial effects may be diluted in patients who underwent non-cardiac surgery [15]. Volatile anesthesia and TIVA have several advantages in various aspects. Therefore, there is still no consensus on which anesthetics regimens is preferable to PPCs in patients undergoing LRS [9].

In the present study, the TIVA group had a shorter LOS than the volatile anesthesia group. A shorter LOS is associated with a reduced risk of opportunistic infections and other adverse events. Moreover, a short LOS reduces medical costs and improves bed turnover rate [16]. Furthermore, a prolonged LOS is associated with surgical procedural factors, patient factors, practical protocols, and the development of perioperative complications [17]. Several studies have demonstrated that there is no difference in LOS between patients in TIVA and volatile anesthesia groups [6, 7]. In a previous meta-analysis, the volatile anesthesia group had a reduced LOS after non-cardiac surgery. However, owing to the limited data on LOS in that meta-analysis, this result should be interpreted with caution [15]. In a retrospective study, the TIVA group had a shorter LOS than the volatile anesthesia group; however, this finding may be attributed to the lower incidence of prolonged air leakage in the TIVA group [9]. As there is no consensus on the superiority of TIVA or volatile anesthesia in LRS, both regimens are considered equivalent choices in the current recommendations for enhanced recovery after surgery (ERAS) programs [18]. Previous studies have indicated that TIVA can improve PONV, postoperative cognitive disorders, and well-being after general anesthesia, which may be beneficial for shortening the LOS after surgery [14, 19–21]. Therefore, increased use of TIVA was reported in a recent study on ERAS programs [22].

The lower incidence of PONV in TIVA group noted in the present study is consistent with the results of previous studies. PONV is an unpleasant postoperative outcome, and is experienced by 20–30% of patients who were placed under general anesthesia during surgery [23]. PONV may cause aspiration of gastric contents, electrolyte imbalance, suture dehiscence, esophageal rupture, and other complications. The use of volatile anesthetics is the significant risk factor of PONV. Besides, the relationship between the use of volatile anesthetics and PONV is dose-dependent [23]. The reduced incidence of PONV after TIVA may lead to better patient satisfaction and earlier recovery after surgery [19].

This study has several strengths. To the best of our knowledge, this is the largest study on the association between different anesthetic regimens and the incidence of PPCs in patients who underwent LRS. In addition, the PSM was used to eliminate the possible effects of confounding factors from the imbalances in collected variables. Moreover, we considered the conflicting results of previous studies and designed this study to provide new evidence regarding the incidence of PPCs in patients who underwent LRS under volatile anesthesia and TIVA, and the results revealed that none of the two regimens is superior to the other.

Our study has several limitations as well. First, owing to the retrospective nature of the study, potential selection bias and influence of unmeasured confounding factors cannot be excluded, though we used PSM to eliminate possible selection bias, and besides, the causality cannot be mentioned. Second, the PSM may narrow down the patient population, but the available sample size is sufficient for the study. Third, the definitions of PPCs in the existing literature vary. We reviewed the existing literature and integrated the common definition of PPCs into our research to ensure that our study is aligned with previous studies. Forth, because of the retrospective study design, the criteria of discharge might be not consistent between different surgeons. Finally, information on the immune statuses of the patients in both groups was not obtained from our hospital database. Analysis of data on immune status may clarify the differences in the immune responses to the TIVA and volatile anesthesia regimens and reveal the role of immunomodulation in postoperative outcomes after LRS.

## Conclusions

In this study, there was no significant difference in the incidence of PPCs between the volatile anesthesia and TIVA groups. The TIVA group had a shorter LOS and lower incidence of PONV than the volatile anesthesia group. Thus, we suggest that TIVA be used for the maintenance of general anesthesia in patients undergoing LRS. However, further prospective studies and randomized controlled trials are needed to elucidate the association between different anesthesia regimens and the occurrence of PPCs after LRS.

### Electronic supplementary material

Below is the link to the electronic supplementary material.

Supplementary Material 1

Supplementary Material 2

## Data Availability

All data are available from the corresponding author on reasonable request.

## Abbreviations

ASA: American Society of Anesthesiologists
BIS: bispectral index
ERAS: enhanced recovery after surgery
HPV: hypoxic pulmonary vasoconstriction
LOS: length of hospital stay
LRS: lung resection surgery
OLV: one-lung ventilation
PONV: postoperative nausea and vomiting
PPC: postoperative pulmonary complications
PSM: propensity score matching
TIVA: total intravenous anesthesia
VATS: video-assisted thoracoscopic surgery
