# Supplementary material for: Total intravenous anesthesia decreases hospital stay but not incidence of postoperative pulmonary complications after lung resection surgery: a propensity score matching study
Source: BMC Anesthesiol. 2023 Oct 17;23:345. doi: 10.1186/s12871-023-02260-4 (PMC10580638; doi:10.1186/s12871-023-02260-4)
Supplement: Supplementary file 1 — Supplementary Material 1 [file 12871_2023_2260_MOESM1_ESM.docx]

Supplementary file 1. The detail of Charlson comorbidity index in the volatile anesthesia and TIVA groups

|  | Original data | | |  | After PSM | | | |
| --- | --- | --- | --- | --- | --- | --- | --- | --- |
|  | TIVA group (n=399) | Volatile anesthesia group (n=732) | *p*-value |  | TIVA group (n=392) | Volatile anesthesia group (n=392) | *p*-value | |
| Age (year) |  |  |  |  |  |  |  | |
| <50 | 93(23.3%) | 128(17.5%) | 0.018 |  | 88 (22.4%) | 84(21.4%) | 0.730 | |
| 50-59 | 102(25.6%) | 188(25.7%) | 0.965 |  | 101(25.8%) | 105(26.8%) | 0.745 | |
| 60-69 | 135(33.8%) | 257(35.1%) | 0.667 |  | 134(34.2%) | 134(34.2%) | 1.000 | |
| 70-70 | 57(14.3%) | 131(17.9%) | 0.119 |  | 57(14.5%) | 57(14.5%) | 1.000 | |
| >80 | 12(3.0%) | 28(3.8%) | 0.477 |  | 12(3.1%) | 12(3.1%) | 1.000 | |
| Myocardial infarction | 18(4.5%) | 23(3.1%) | 0.239 |  | 18(4.6%) | 10(2.6%) | 0.124 | |
| congestive heart failure | 5(1.3%) | 6(0.8%) | 0.478 |  | 5(1.3%) | 3(0.8%) | 0.477 | |
| Peripheral vascular disease | 3(0.8%) | 3(0.4%) | 0.449 |  | 3(0.8%) | 2(0.5%) | 0.654 | |
| CVA or TIA | 9(2.3%) | 10(1.4%) | 0.266 |  | 9(2.3%) | 4(1.0%) | 0.162 | |
| Dementia | 0 | 1(0.1%) | 0.460 |  | 0 | 0 | - | |
| COPD | 7(1.8%) | 15(2.0%) | 0.732 |  | 7(1.8%) | 7(1.8%) | 1.000 | |
| Connective tissue disease | 9(2.3%) | 21(2.9%) | 0.540 |  | 9(2.3%) | 13(3.3%) | 0.387 | |
| Peptic ulcer disease | 21(5.3%) | 28(3.8%) | 0.256 |  | 21(5.4%) | 16(4.1%) | 0.400 | |
| Liver disease |  |  |  |  |  |  |  | |
| None | 393(98.5%) | 720(98.4%) | 0.862 |  | 386(98.5%) | 386(98.5%) | 1.000 | |
| Mild | 2(0.5%) | 9(1.2%) | 0.233 |  | 2(0.5%) | 4(1.0%) | 0.412 | |
| Moderate to severe | 4(1.0%) | 3(0.4%) | 0.225 |  | 4(1.0%) | 2(0.5%) | 0.412 | |
| DM |  |  |  |  |  |  |  | |
| None or diet-controlled | 353(88.5%) | 639(87.3%) | 0.565 |  | 346(88.3%) | 357(91.1%) | 0.197 | |
| Uncomplicated | 46(11.5%) | 92(12.6%) | 0.610 |  | 46(11.7%) | 35(8.9%) | 0.197 | |
| End-organ damage | 0 | 1(0.1%) | 0.460 |  | 0 | 0 | - | |
| Hemiplegia | 0 | 1(0.1%) | 0.460 |  | 0 | 0 | - | |
| Moderate to severe CKD | 0 | 7(1.0%) | 0.050 |  | 0 | 4(1.0%) | 0.045 | |
| Solid tumor |  |  |  |  |  |  |  | |
| None | 44(11.0%) | 71(9.7%) | 0.480 |  | 42(10.7%) | 47(12.0%) | 0.573 | |
| Localized | 286(71.7%) | 526(71.9%) | 0.949 |  | 284(72.4%) | 279(71.2%) | 0.691 | |
| Metastatic | 69(17.3%) | 135(18.4%) | 0.631 |  | 66(16.8%) | 66(16.8%) | 1.000 | |
| Leukemia | 1(0.3%) | 2(0.3%) | 0.944 |  | 1(0.3%) | 1(0.3%) | 1.000 | |
| Lymphoma | 0 | 2(0.3%) | 0.296 |  | 0 | 1(0.3%) | 0.317 | |
| AIDS | 0 | 0 |  |  | 0 | 0 |  | |
| Values are presented as counts (percent)  Abbreviations: AIDS, acquired Immunodeficiency syndrome; CKD, chronic kidney disease; COPD, chronic obstructive pulmonary disease; CVA, cerebrovascular accident; DM, diabetes mellitus; PSM, propensity score matching; TIA, transient ischemic attack; TIVA, total intravenous anesthesia | | | | | | | |  |
