# Supplementary figures and images for: Total intravenous anesthesia decreases hospital stay but not incidence of postoperative pulmonary complications after lung resection surgery: a propensity score matching study
Source: BMC Anesthesiol. 2023 Oct 17;23:345. doi: 10.1186/s12871-023-02260-4 (PMC10580638; doi:10.1186/s12871-023-02260-4)

Supplementary file 2. The distribution of propensity scores


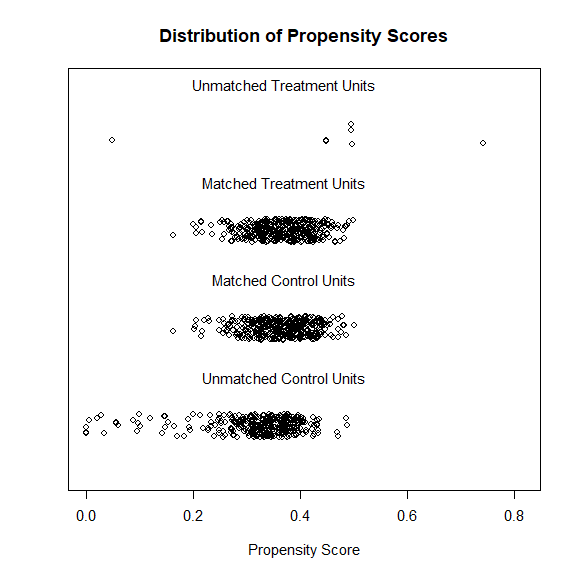

Supplement: Supplementary file 2 — Supplementary Material 2 [file 12871_2023_2260_MOESM2_ESM.docx]
